# Sex-lethal in neurons controls female body growth in *Drosophila*

**DOI:** 10.1080/19336934.2018.1502535

**Published:** 2018-08-21

**Authors:** Annick Sawala, Alex P. Gould

**Affiliations:** Physiology & Metabolism Laboratory, The Francis Crick Institute, London, UK

**Keywords:** Drosophila, growth, sexual differentiation, sexual size dimorphism, Sex-lethal, insulin signaling

## Abstract

Sexual size dimorphism (SSD), a sex difference in body size, is widespread throughout the animal kingdom, raising the question of how sex influences existing growth regulatory pathways to bring about SSD. In insects, somatic sexual differentiation has long been considered to be controlled strictly cell-autonomously. Here, we discuss our surprising finding that in *Drosophila* larvae, the sex determination gene *Sex-lethal* (*Sxl*) functions in neurons to non-autonomously specify SSD. We found that Sxl is required in specific neuronal subsets to upregulate female body growth, including in the neurosecretory insulin producing cells, even though insulin-like peptides themselves appear not to be involved. SSD regulation by neuronal Sxl is also independent of its known splicing targets, *transformer* and *msl-2*, suggesting that it involves a new molecular mechanism. Interestingly, SSD control by neuronal Sxl is selective for larval, not imaginal tissue types, and operates in addition to cell-autonomous effects of Sxl and Tra, which are present in both larval and imaginal tissues. Overall, our findings add to a small but growing number of studies reporting non-autonomous, likely hormonal, control of sex differences in *Drosophila*, and suggest that the principles of sexual differentiation in insects and mammals may be more similar than previously thought.

Sex differences in body size, termed sexual size dimorphisms (SSD), are widespread throughout the animal kingdom. Although the mechanisms by which nutrition and other environmental factors regulate growth have been widely studied, much less is known about how the sex of an organism influences its growth. This is particularly true in the fruit fly *Drosophila melanogaster*, a key model organism for studying growth regulation, where females are ~ 30% larger than males.

In *Drosophila*, the presence of two X chromosomes in females activates expression, in the early embryo, of the sex determination gene *Sex-lethal* (*Sxl*), which is thereafter maintained via positive autoregulation [1–3]. *Sxl* encodes an RNA binding protein that regulates sexual differentiation via *transformer* (*tra*) splicing and also X-chromosome dosage compensation by regulating the splicing and translation of *male-specific lethal 2* (*msl-2*) [4–6]. It has recently been suggested that SSD may be regulated, in part, by a *Sxl* independent mechanism [7], but our recent data indicates that loss of *Sxl* alone completely masculinizes female body size [8]. The nature of the genes and physiological processes that lie downstream of *Sxl* to control SSD is, however, still very unclear. The classic textbook view is that somatic sex determination in *Drosophila* operates in a strictly autonomous manner --- where every cell needs to know its sex [9,10]. Here, we will discuss our recent discovery that, in contrast to this view, *Sxl* acts in specific neurons in the larval brain to promote female larval body growth in a non-cell autonomous manner [8].

## Sxl acts in neurons to remotely control SSD via a novel mechanism

A key finding from our study is that *Sxl* functions in the brain to remotely increase female body growth [8]. Hence, RNAi knockdown of *Sxl* with a neuron-specific Gal4 driver was observed to decrease the body size of females to that of males. Furthermore, we found that restoring *Sxl* expression in neurons alone rescues female body size in a *Sxl* mutant background, although not as completely as did a complete copy of the gene. The reasons for incomplete rescue may be technical (e.g. levels/timing of transgene expression and/or the fact that the *UAS-Sxl* transgene only encodes a subset of *Sxl* isoforms [11,12]) or perhaps due to additional cell-autonomous actions of *Sxl* (see below). Nevertheless, the data strongly support the surprising conclusion that neuronal Sxl can enhance the growth of the female larval body.

Interestingly, using the same Gal4 tools to manipulate the *Sxl* splicing target *tra* in neurons does not show comparable effects on SSD. Furthermore, we found that neuronal expression of TraF, the female splice-variant of *tra* that feminizes male neurons [13,14], does not increase body size in males. Overall, these findings suggest that neuronal Sxl promotes female body growth independently of *tra*, consistent with a longstanding suggestion that SSD regulation is *tra*-independent [3,4,11]. However, several recent studies have reported that whole body *tra* mutants do show a reduced female body size when measured either as larval body mass [7] or as pupal volume [15]. This mutant phenotype may be due to cell- or tissue-autonomous effects of *Sxl* and *tra* that have been observed in several tissues and appear to operate in addition to the non-autonomous role of Sxl in neurons [8,15–17]. It has also been reported that *tra* activity in the larval fat body non-autonomously increases female body growth [15]. In our hands, we were unable to detect similar non-autonomous functions for either *Sxl* or *tra* manipulations in the fat body, suggesting these may depend on very specific laboratory or genetic background conditions. Interestingly, the adult fat body has also been implicated in non-cell autonomous regulation of sex-specific behavior. Sexually dimorphic gene expression in the adult head fat body, under the control of the Sxl/Tra pathway, regulates secreted factors that interact with brain circuits to promote male-specific courtship behavior [18–20]. One of the secreted factors is Takeout, a protein that may function as a carrier for juvenile hormone, which is important for male courtship behavior [19–21].

Our data indicate that the role of neuronal Sxl in SSD regulation is also independent of its other major target, *male-specific lethal-2 (msl-2*.) Msl-2 is required for assembly of the dosage compensation complex on the male X chromosome, which induces hypertranscription of X-linked genes and is translationally repressed by Sxl in females [22–26]. As expected, we observed that neuronal Sxl knockdown leads to ectopic upregulation of Msl-2 protein in females and induces X-chromosome hyperactivation [8]. Theoretically, this female misexpression of Msl-2 could cause the observed reduction in female body size, either because of an endogenous function of Msl-2 as a growth inhibitor or because of ‘sickness’ effects due to upsets in dosage compensation. However, we were able to rule out both of these possibilities for two reasons. Firstly, ectopic neuronal Msl-2 expression via a *UAS-msl-2* transgene is not sufficient to decrease female body size and conversely, neuronal *msl-2* knockdown cannot increase male body size. And secondly, knockdown of msl-2 in a *Sxl* RNAi context rescues ectopic X-chromosome hyperactivation but does not rescue female body size.

Taken together, our data suggest that the mechanism of SSD regulation by neuronal Sxl does not involve the same molecular pathways that control other aspects of sexual differentiation or dosage compensation. This suggests that Sxl may regulate an as yet unidentified mRNA target to control SSD. A small number of other mRNAs have been identified as actual or possible direct targets of Sxl in different adult tissues [27–31]. A potentially interesting new target is *found in neurons* (*fne*) [31], which is expressed in neurons and encodes an RNA-binding protein of the Elav/Hu family, a group of evolutionarily conserved proteins that are closely related to Sxl and show similar mRNA target specificity [32]. The biological consequences of *fne* regulation by Sxl are poorly understood and, in particular, have not been analysed in specific neurons or in the larval brain. However, *fne* null mutants have defects in male courtship behavior [33], suggesting that *fne* could play a specific role in sexual dimorphism. In addition to its role in RNA regulation, Sxl has also been found to interact with cytoplasmic components of the Hedgehog signaling pathway [34], and a role in regulating stability and nuclear localization of the transcription factor Cubitus interruptus has also been reported [35]. Furthermore, Sxl binds a subunit of RNA polymerase III, suggesting that it may regulate transcription [36]. Given the variety of mechanisms by which Sxl could potentially regulate its targets, the dissection of the molecular player(s) directly downstream of Sxl in the control of SSD is likely to be a complex undertaking requiring unbiased genome-wide approaches.

## SSD is regulated differently in larval and imaginal tissues

**Figure 1.**
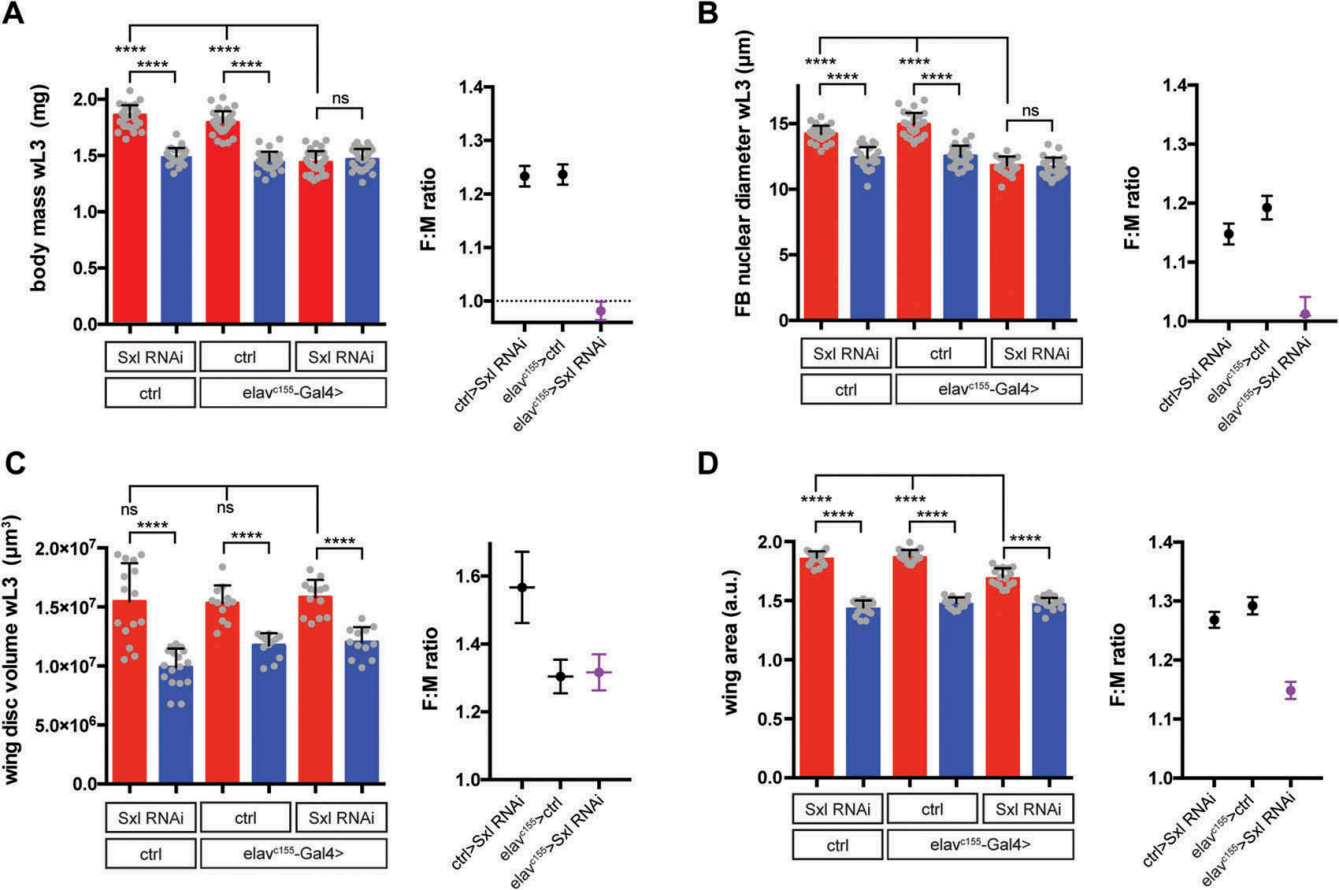
**Effect of neuronal knockdown of Sxl on sexual size dimorphism in wandering L3 (wL3) larvae and adults**. For each panel, bar graphs on the left show size measurements in males (blue) and females (red), with individual data points plotted as grey dots, error bars show SD. Right graphs plot female to male ratios (+ SEM) as a measure of sexual size dimorphism (SSD). Pan-neuronal expression of Sxl RNAi (elav^c155^> Sxl RNAi) abolishes SSD at the level of larval body mass **(A)** and fat body nuclear diameter **(B**), whereas SSD in the wing imaginal disc remains intact in wL3 larvae **(C)**. Although neuronal Sxl knockdown does not affect wing imaginal disc SSD during larval stages, SSD of the adult wing is reduced **(D)**. ****P < 0.0001 according to One-way ANOVA with multiple comparison correction. Methods: Early L1 (first instar) larvae of all genotypes were raised on standard yeast-glucose-cornmeal medium at a fixed density at 29°C until the wandering L3 stage. For adult samples, animals were incubated at 18°C during pupal stages to maximise survival and adults were analysed 1–2 days after eclosion. Larval body mass was measured for individual larvae on a microbalance. Fat body nuclear diameter and wing imaginal disc volume were measured from DAPI-stained, fixed tissues imaged by confocal microscopy. Adult wings were imaged by light microscopy and area measurements were performed using a custom pipeline in CellProfiler [58]. Detailed methods are provided in [8].

Our finding that sex differences in body size are controlled by Sxl acting in the brain contradicts the longstanding dogma that the *Drosophila* sexual differentiation pathway in the soma acts strictly cell-autonomously. In the case of SSD, evidence for such a cell-autonomous mechanism came from early studies of gynandromorphs (mosaic male/female animals), which clearly showed that female structures are larger than their male counterparts within the same adult fly [37]. We think that resolution of the apparent discrepancy lies, at least in part, in our surprising finding that the neuronal Sxl mechanism only controls SSD of larval polyploid tissues, not the imaginal discs that form the adult structures analyzed in the gynandromorphs. Our study [8] was focused on sex differences in larval body mass, the bulk of which is composed of larval polyploid tissues. Indeed, when we looked at sex differences in the size of an individual larval tissue, the fat body, we found that Sxl knockdown entirely abolished SSD (Figure 1B). By contrast, SSD in the wing imaginal discs from the same wandering L3 larvae remained intact (Figure 1C). This demonstrates that SSD is regulated differently in larval versus imaginal tissue types. It is important to note that we and others [8,15,17,38] have shown that Sxl and Tra have additional cell/tissue-autonomous effects on growth in both tissue types and, in imaginal tissues, these likely explain the gynandromorph observations. Such a combination of cell-autonomous and non-autonomous (hormonal) mechanisms is not restricted to flies and is also important for SSD in mammals [39].

**Figure 2.**
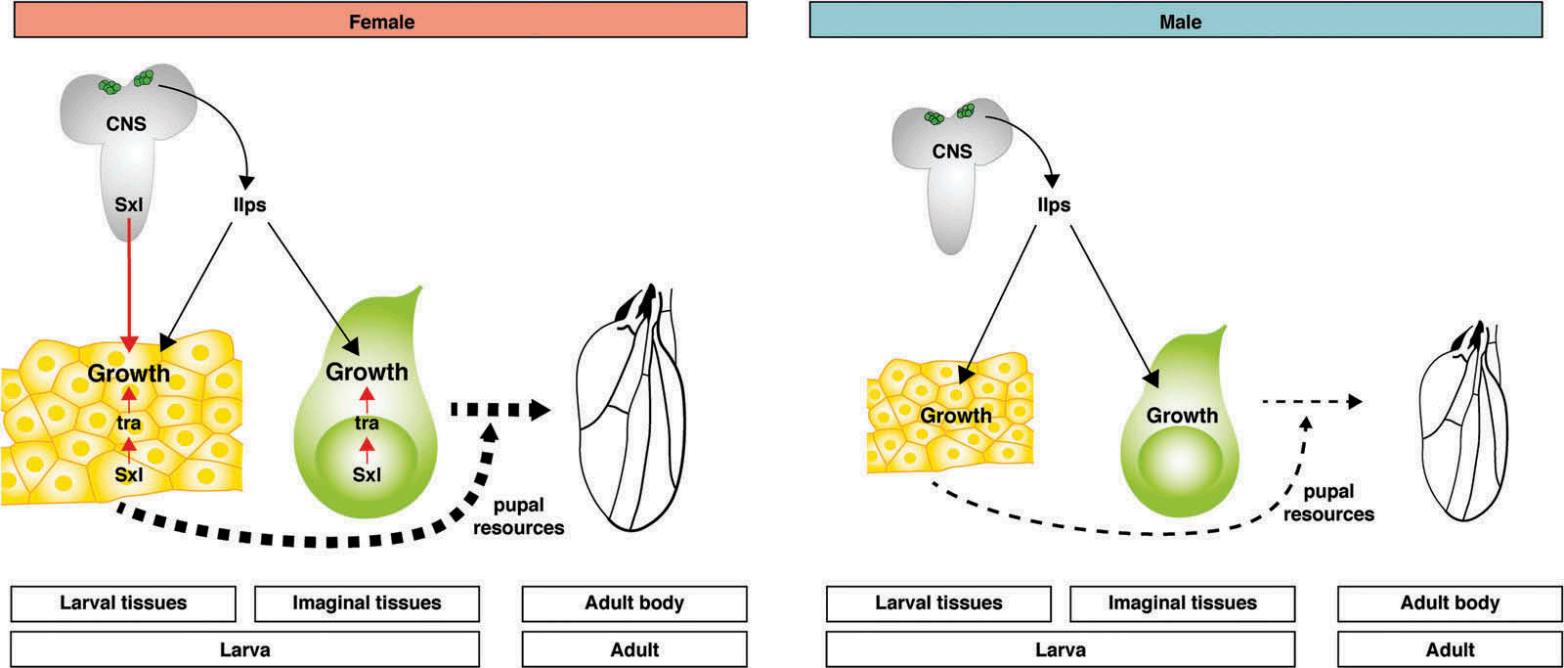
**A relay model for the neuronal control of SSD in *Drosophila***. In both larval and imaginal tissues, Sxl acts cell-autonomously via Tra to increase female growth. Additionally, neuronal *Sxl* activity in the larval brain remotely increases the growth of larval tissues in females, in a pathway that is largely independent of Tra and of insulin signalling via IPC-secreted Ilps, which promotes growth in a non-sex specific manner. Although imaginal tissue growth at larval stages is unaffected by neuronal Sxl, the increased female size of larval tissues provides a larger store of pupal resources (nutrients and/or signalling molecules) to promote/maintain increased female growth of imaginal tissues during non-feeding pupal stages. This model summarises the results from our study and does not yet incorporate results from other studies [7,15] reporting effects of Myc or fat body Tra on systemic growth.

**Figure 3.**
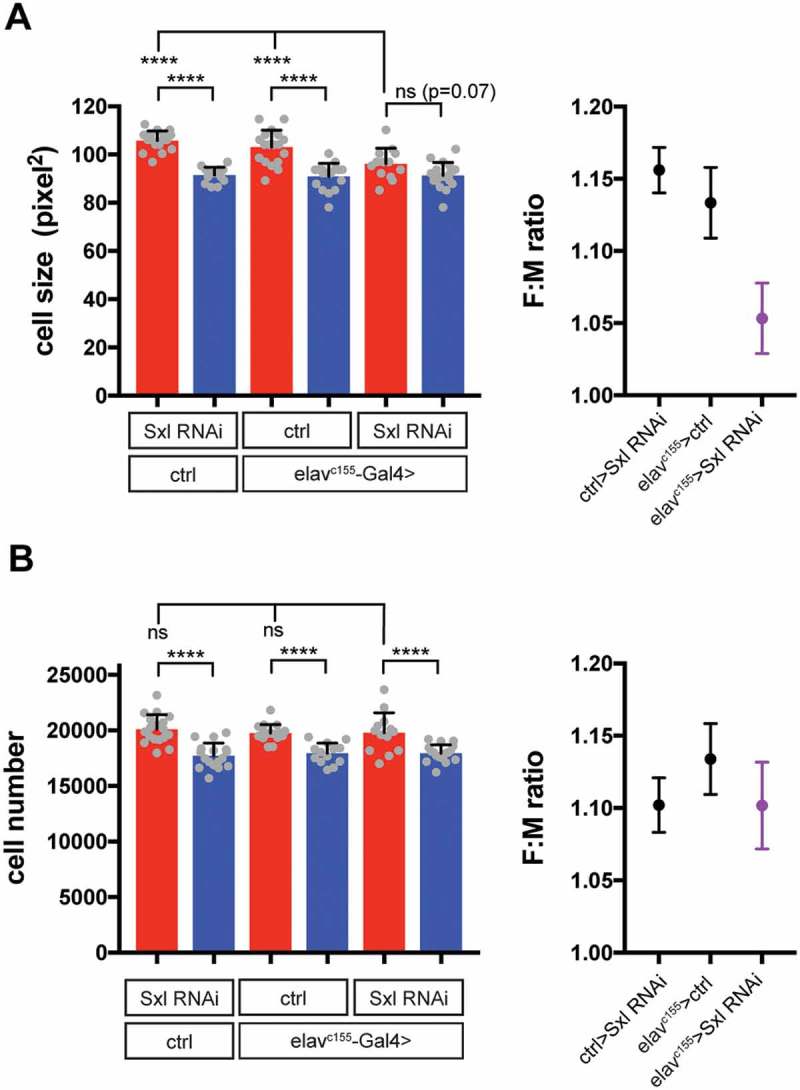
**Neuronal Sxl increases adult wing size in females via an effect on cell size, not cell number**. Neuronal Sxl knockdown (*elav^c155^> Sxl RNAi*) was performed at 29°C during larval stages, followed by a shift to 18°C until eclosion to reduce *elav^c155^-Gal4* activity during pupal stages (for details, see legend to Figure 1 and [8]). Trichome density (as a measure of cell density) in the adult wing was analysed manually in a region of interest in the wing region lined by longitudinal veins 4 and 5 and the posterior cross vein. **(A)** Cell size in the adult wing was estimated as the inverse of trichome density. Neuronal knockdown of Sxl strongly reduced cell size in females, nearly to the size seen in males. **(B)** Cell number in the adult wing was estimated as the product of wing area and trichome density for each wing. Neuronal Sxl knockdown had no effect on female cell number. For each panel, bar graphs on the left show size measurements in males (blue) and females (red), with individual data points plotted as grey dots, error bars show SD. Right graphs plot female to male ratios (+ SEM) as a measure of sexual size dimorphism (SSD). ****P < 0.0001 according to One-way ANOVA with multiple comparison correction.

Given that neuronal Sxl does not seem to affect SSD in imaginal discs when they are within the larva, can it nevertheless influence SSD in their derivatives, the body of the adult fly? Our data reveals that driving Sxl RNAi in neurons does indeed decrease adult body mass and wing size in females, although some SSD remains in both cases ([8] and Figure 1D). We therefore propose that, by increasing the growth of larval tissues in females, neuronal Sxl provides females with increased pupal resources to promote or maintain SSD in imaginal discs during metamorphosis (Figure 2). Increased pupal resources could equate to nutrients released from histolyzed larval tissues to fuel imaginal growth and/or to pupal growth factors. An alternative possibility is that neuronal Sxl acts specifically during pupal stages to increase imaginal tissue size in females. We have now analyzed more closely the effect of neuronal Sxl knockdown on the growth of the adult wing. Consistent with previous findings [40,41], female wing size is larger than that of male flies due to an increase in both cell number and cell size (Figure 3). Interestingly, loss of neuronal Sxl strongly decreases the sex difference in adult wing cell size, while the sex difference in adult wing cell number remains intact (Figure 3). These results suggest that neuronal Sxl influences the size of adult imaginal structures via an effect on cell growth not cell proliferation, just as it does for larval polyploid tissues.

The selective effect of neuronal Sxl on polyploid tissues during the larval period is intriguing and, to our knowledge, it is the first systemic growth signal identified to act specifically on the larval polyploid tissues of *Drosophila*. Perhaps this differential mechanism of SSD regulation reflects the different modes of growth for larval and imaginal tissues. Over the time period that SSD arises (second and early third larval instars), larval polyploid tissues grow entirely via an increase in cell size driven by endoreplication, while imaginal disc cells are diploid and grow through cell proliferation with no accompanying increase in cell size [42,43]. It should be noted that we measured fat body SSD at the level of nuclear diameter but we have not yet distinguished whether or not the sex difference in nuclear (and presumably cell) size correlates with a change in endoreplication. However, identification of a mechanism that specifically targets cell growth rather than proliferation is of great interest. Also, it is known that many cancers undergo endoreplication, which confers increased cancer cell growth/survival and resistance to anti-mitotic drugs [44].

## The role of insulin signaling in SSD regulation

A key question arising from our study is how the activity of Sxl in neurons is relayed to polyploid tissues throughout the larva to stimulate their growth. Ultimately, the mechanism is likely to involve a factor secreted from the neurons in which Sxl functions or from the neuronal or non-neuronal cells to which they project. A Gal4 driver screen was performed to identify the neurons in which Sxl functions to promote SSD [8]. We isolated several broadly expressed peptidergic and GABAergic neuronal drivers, as well as drivers expressed specifically in the insulin producing cells (IPCs), a cluster of 7 neurosecretory neurons in the *pars intercerebralis* of the brain which secrete Insulin-like peptides (Ilps2, 3, 5) into the circulation. The effect of the broad peptidergic drivers and one of the two GABAergic drivers could be explained partially if not fully by their activity in the IPCs. However, the ‘GABAergic’ Gad1-Gal4 driver is not expressed in IPCs, suggesting that Sxl acts in at least two non-overlapping sets of neurons: Gad1-neurons and IPCs. The identification of these two sets of neurons immediately suggests two potential signals that could be released into the circulation to promote larval SSD: Ilps, which are key regulators of growth [45,46], and GABA, an inhibitory neurotransmitter used widely within the brain but which can also be released into the hemolymph from a set of peptidergic neurons [47]. So far, we have no evidence for GABA being involved in SSD, although a more careful study may be required to conclusively rule out a role for this neurotransmitter. To complicate matters, although Gad1-neurons have been described as ‘GABAergic’, the overlap with GABA expression is only partial [8], so it is not even clear if GABA-producing neurons are involved.

The question of whether insulin like peptides (Ilps) mediate the SSD function of IPCs is a complex one. Sex differences in Insulin-like growth factor 1 (IGF-1) production are known to be important for SSD in mammals [48,49]. By analogy, an attractive model for SSD regulation in *Drosophila* would be that Sxl in IPCs promotes the release of Ilps into the larval hemolymph, leading to higher levels of insulin signaling in peripheral tissues and stimulating female growth rate. However, at the time when sex differences in growth rate are maximal (second and early third instar larvae), we were unable to detect a sex difference in Ilp release from IPCs or in the activation of peripheral insulin signaling [8]. Furthermore, manipulating insulin production or secretion in IPCs had no effects on larval body SSD [8]. Finally, null mutants for Ilp2 or Ilps1-5, which lack all Ilps normally expressed in IPCs, show a strong reduction in body mass but SSD remains intact [8]. Thus, surprisingly, our data suggests that larval body SSD is dependent upon Sxl in IPCs, yet independent of Ilps. There is, nevertheless, evidence that insulin signaling likely plays a late and imaginal-tissue specific role in *Drosophila* SSD. In the late third instar larva, higher levels of Ilp2 secretion and insulin signaling have been detected in females [15]. Furthermore, hypomorphic mutant alleles of the insulin receptor cause not only a dramatic reduction in adult body mass but also strongly reduce SSD [50]. These findings suggest that insulin signaling may be important for SSD in imaginal tissues during later larval stages, when polyploid tissues have essentially ceased growth. In support of this, we found that experimentally reducing IPC size, which is thought to decrease insulin production, abolished SSD in the wing imaginal disc, even though SSD at the level of larval body mass remained intact [8].

## Outlook

Many interesting issues remain regarding the molecular mechanisms by which neuronal Sxl relays SSD information to peripheral tissues. These include the molecular targets of neuronal Sxl, the complexity of the SSD neuronal circuitry, the nature of the signal that relays neuronal Sxl status from the brain to the periphery and how it is specific for larval polyploid tissues. These issues may well be rather fly-centric but investigating them promises to shed light on generally important topics such as endoreplicative versus proliferative growth, brain regulation of body size and sex-specific differences in metabolism.

Our central finding that SSD regulation involves a non-autonomous, likely hormonal mechanism adds to a small but growing number of findings from *Drosophila* that indicate that sex differences in growth, physiology and behavior are regulated via secreted factors, which may act as sex hormones [15,19–21,51–53]. This raises the possibility that even though the primary sex determination signals are diverse and rapidly evolving across metazoans, some of the downstream mechanisms that control sexually dimorphic physiology may be more conserved. Perhaps this is not an unreasonable proposition considering that certain fundamental differences between the sexes exist in almost all animals, such as a higher female metabolic cost of reproduction [54]. Likely, these sex-specific physiologies also contribute to the widely observed sex differences in human disease risk, presentation and drug responses [55–57]. Applying the powerful genetics of *Drosophila* to reveal molecular underpinnings of sexually dimorphic phenotypes may help our understanding of this important new area of biomedical research.

## Abbreviations

SSD: Sexual size dimorphism
Sxl: Sex-lethal
tra: transformer
msl-2: male-specific lethal-2
fne: found in neurons
IPC: insulin producing cell
Ilp: insulin-like peptide
GABA: gamma-aminobutyric acid
IGF-1: insulin-like growth factor-1
